# Immunomodulatory Effects of Green Tea Polyphenols †

**DOI:** 10.3390/molecules26123755

**Published:** 2021-06-20

**Authors:** Shuzhen Wang, Zhiliang Li, Yuting Ma, Yan Liu, Chi-Chen Lin, Shiming Li, Jianfeng Zhan, Chi-Tang Ho

**Affiliations:** 1 Hubei Collaborative Innovation Center for the Characteristic Resources Exploitation of Dabie Mountains, Hubei Zhongke Research Institute of Industrial Technology, College of Life Science, Huanggang Normal University, Huanggang 438000, China; wangshuzhen710@whu.edu.cn (S.W.); swlzl@hgnu.edu.cn (Z.L.); 2019230240127@smail.hgnu.edu.cn (Y.M.); 2019230240405@smail.hgnu.edu.cn (Y.L.); swzjf@hgnu.edu.cn (J.Z.); 2 Institute of Biomedical Science, The iEGG and Animal Biotechnology Center, National Chung-Hsing University, Taichung 402, Taiwan; lincc@dragon.nchu.edu.tw; 3 Department of Food Science, Rutgers University, New Brunswick, NJ 08901, USA

**Keywords:** green tea polyphenols, immunomodulatory, autoimmune diseases, anti-inflammatory action, epigallocatechin-3-gallate (EGCG)

## Abstract

Green tea and its bioactive components, especially polyphenols, possess many health-promoting and disease-preventing benefits, especially anti-inflammatory, antioxidant, anticancer, and metabolic modulation effects with multi-target modes of action. However, the effect of tea polyphenols on immune function has not been well studied. Moreover, the underlying cellular and molecular mechanisms mediating immunoregulation are not well understood. This review summarizes the recent studies on the immune-potentiating effects and corresponding mechanisms of tea polyphenols, especially the main components of (–)-epigallocatechin-3-gallate (EGCG) and (–)-epicatechin-3-gallate (ECG). In addition, the benefits towards immune-related diseases, such as autoimmune diseases, cutaneous-related immune diseases, and obesity-related immune diseases, have been discussed.

## 1. Introduction

The human immune system has developed defensive components possessing functions specialized for mucosal areas, such as mucus and its constituents, secretory immunoglobulins, and unique subsets of leukocytes localizing to or maturating in mucosal regions [1]. Mucosal surfaces are the interfaces between the external and internal environments, through which gases, nutrients, waste products, and other materials can move [2]. At the same time, mucosal surfaces also provide ideal sites for the entry of pathogens. Immunomodulation encompasses immunostimulation or immunoinhibition of certain cellular and/or humoral immune responses. These defensive components prevent attachment and entry of pathogens to host tissues and mobilize other immune components after the entry of microbes, which ultimately resist against particular pathogens or foreign substances [1]. Particularly, the sophisticated immune response relies on the reactions of a number of germline-encoded pattern-recognition receptors (PRRs) with conserved pathogenic structures of pathogen-associated molecular patterns (PAMPs) [3,4].

The most extensively studied PRRs is the Toll-like receptor (TLR) family, which can be further divided into several subfamilies based on recognized ligands: TLRs 1, 2, and 6 identifying lipopeptides and glycolipids; TLRs 7, 8, and 9 recognizing nucleic acids (ssRNA and unmethylated CpG); TLR3 distinguishing dsRNA associated with viral infection; TLR4 recognizing fibronectin, lipopolysaccharides (LPS), and heat shock proteins; TLR5 recognizing bacterial flagellin; TLRs 11 and 12 recognizing profilin and actin-binding protein (Table 1) [5]. TLRs are expressed by a variety of immune cells, including T cells, B cells, dendritic cells (DCs), and natural killer (NK) cells. NK cells express high levels of TLRs 1, 3 and 6 [6,7]. DCs express TLRs 2, 4, 7, and 9 [8]. Human peripheral blood T cells express TLRs 1–5, 7, and 9 [9]. B cells mainly express TLRs 3, 4, and 9 [10]. In particular, TLR2 binds to a wider range of ligands in recognizing bacterial, viral, fungal, and endogenous substances. Moreover, dimerization of TLR2 with TLR 6 or TLR1 is vital for identifying bacterial lipoproteins and lipopeptides [11]. TLR 2 serves as crucial targets for immunotherapy, especially in malignant diseases [12]. Once activated TLR 2, there is an increase in the nuclear transcription factor-κB (NF-κB), which induces expression of cytokines to enhance immunity. In humans, TLR 4 specifically recognizes bacteria LPS, endogenous molecules produced during tissue damage, and other pathogen components [13].

The activation of TLRs with corresponding ligands could affect myeloid and lymphoid progenitors, and trigger signaling cascades leading to production of pro-inflammatory cytokines and chemokines. After pathogen recognition, the TLR signal transduction is initiated through the toll/interleukin 1 receptor (TIR) domain [5]. Most TLRs use a TIR-containing adaptor MyD88 to trigger a certain signaling pathway, in which activated NF-κB induce expression of critical genes for inflammatory cytokines [14]. MyD88, a downstream adaptor protein of mammalian TLR and Interleukin (IL)-1 receptor (IL-1R) families, can link TLRs to IL-1R associated kinases (IRAKs), leading to the activation of NF-κB, activator protein 1, and MAPKs [14]. Therefore, MyD88 is a critical player in inflammatory signaling pathways. The dysregulated activity of TLR is often associated with high risk of chronic inflammatory and immune diseases, such as aging, immunosenescence, and even autoimmune diseases including diabetes, inflammatory bowel disease, rheumatoid arthritis, hepatitis, and systemic lupus erythematosus [15,16,17].

**Figure 1:**
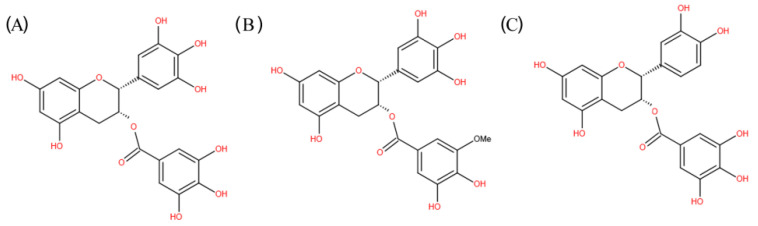
Chemical structure of EGCG (**A**), EGCG3’’Me (**B**), and ECG (**C**).

Natural products and their derivatives exert immunomodulatory effects on chronic diseases through stimulating various branches and components of the immune system, and are promising as anti-oxidative, antimicrobial, regulation of metabolism, anti-inflammatory, anticancer, as well as cardiovascular protective and neurological protective agents [5,18]. In particular, natural products, such as herbal medicines, probiotics, vitamins, polyphenols, and fatty acids, have been demonstrated to possess immunomodulatory actions [19]. Today, about 70% of drugs on the market originate directly or indirectly from natural sources [20]. Many phytochemicals exert their action by targeting TLRs and their signaling molecules downstream. In particular, green tea, obtained from the leaves of *Camellia sinensis*, possesses important medicinal value. The green tea polyphenols (GTPs), especially (–)-epigallocatechin-3-gallate (EGCG) and (–)-epicatechingallate (ECG) (Figure 1), can greatly enhance immune response and lower the risk of inflammation and immune-related diseases [21,22].

Health-promoting multifunctionality has been well illustrated for tea polyphenols, especially anti-inflammatory, antioxidant, anticancer effects, and metabolic modulation with multi-target action [23]. As natural immune modulators, tea polyphenols could control and defeat disorders affecting immune system through up- or down-regulating immune responses without undesired adverse effects [23]. In the present review, the advancements in recent 30 years for utilizing green tea polyphenols for immunomodulation, as well as their molecular and cellular mechanisms to modulate inflammation, have been summarized. Moreover, important molecular targets of green tea polyphenols have also been clarified, hoping to bring broad application of green tea as functional foods.

## 2. Immune Potentiating Effects of Tea Polyphenols

GTPs and their derivatives act through stimulating multiple TLR signaling pathways in human and could effectively inhibit proliferation of murine lymphocytes (Table 2) [24]. EGCG, comprising approximate 55–70% of total tea polyphenols, exerted immunomodulatory effects on tumor immunity in different pre-clinical models, and was exploited for treatment of chemo-insensitive but immunologically responsive tumors [25]. Moreover, EGCG inhibited IKKβ, a downstream signaling molecular of MyD88-dependent pathway [26]. Furthermore, EGCG also inhibited NF-κB activation induced by TLR4 and TLR2 agonists [26]. EGCG could prevent the production of interferon gamma (IFN*γ*) by SEB (*Staphylococcus aureus* enterotoxin B)-stimulated peripheral blood mononuclear cells [27]. Moreover, EGCG could reduce the depletion of antigen-presenting cells (APCs), and prevent ultraviolet-induced immunosuppression [28,29]. In vitro, EGCG could suppress the maturation of mouse bone marrow-derived and human monocytes-derived DCs, which were the main APCs [29,30,31]. In invertebrate Kuruma shrimp *Marsupeneaus japonicus* challenged with white spot syndrome virus (WSSV) and the Gram-negative bacterium *Vibrio alginolyticus*, EGCG pretreatment significantly delayed and reduced mortality through regulating expression of innate immune-related genes, such as *IMD*, *proPO*, *QM*, *myosin*, *Rho*, *Rab7*, *p53*, *TNF-*α, *MAPK*, and *NOS* [32]. In the shrimp innate immune system, EGCG could serve as an enhancer of immune parameters (total hemocyte count, phenoloxidase, and superoxide dismutase activities) and an inhibitor of apoptosis, finally delaying or even reducing mortality upon pathogen challenge [32].

The Th1 and Th2 are critical members in cell- and humoral-mediated immunity, respectively. In the body, Th1/Th2 keeps a dynamic balance, which is vital for maintaining sound immune function. GTPs partially reduced IFNγ-induced phosphorylation and function of STAT1, prevented upregulation of inducible nitric oxide synthetase (iNOS) 2 and NF-κB, and inhibited transcription and secretion of TNF-α and IFNγ, all inferring that GTPs may preferentially inhibit Th1-like T cell immunity [33,34,35]. GTPs might also inhibit these intracellular signals, which are involved in T cell activation and differentiation into Th1-like effector cells. GTPs supplementation could elevate the anti-inflammatory cytokine IL-4, which is necessary for the proliferation and activation of Th2 cell [36]. Dietary GTPs could decrease the ratio of IFNγ/IL-4, an index of Th1/Th2 [36].

### 2.1. Effects on Cellular Immunity

T cells could release various cytokines, stimulate B cell activation and proliferation, and improve immune response [37]. The CD4+ T cells participate in recognizing antigens presented by MHC type II (exogenousAg), and CD8+ T cells participate in the recognition of antigens presented by MHC type I (endogenous Ag). The ratio of CD4+/CD8+ (<1) refers to a sign of immunodeficiency [37]. Dietary GTPs enhance defense ability of the host against exogenous infection through promoting immune cell proliferation, activating T lymphocytes, elevating the percentage of CD4+T cell, elevating the ratio of CD4+/CD8+, improving T lymphocyte transformation (LTT), and recovering cells from immune damage caused by oxidative stress in piglets [36]. In mixed lymphocyte reactions, GTPs could effectively inhibit IFN*γ* secretion by cultured monoclonal T cells and alloreactive T cells, and the inhibitory effects in vitro were routinely notable at 3–20 µg/mL [38]. Oral GTPs could significantly prolong the minor antigen-disparate skin graft survival, significantly induce apoptosis of T cells and APCs, as well as greatly decrease the frequency of donor-reactive IFNγ-producing T cells in recipient secondary lymphoid organs [38]. However, oral GTPs could not alter DCs trafficking to lymph nodes or affect metalloproteinase activity in the skin graft [38]. In addition, GTPs could influence both the expression of T-cell receptor complex and antigen-specific T-cell responses [39].

EGCG (2.5–15 mM) could inhibit T cell division and cycle progression in a dose-dependent manner, and the inhibitory effect was more pronounced in CD4+ T cells than CD8+ T cells [38]. Compared with CD8+ T cells, CD4+ T cells were more responsive to EGCG [38]. At a physiologically relevant concentrations (2.5–10 mmol/L), EGCG inhibited splenocyte proliferation stimulated by T cell mitogen concanavalin A (Con A) in a dose-dependent manner [40]. Moreover, T cell division and cell cycle progression could also be inhibited by EGCG [40]. EGCG could also suppress the LPS-induced phenotypic and functional maturation of murine DCs through inhibiting the mitogen-activated protein kinases (MAPK) and NF-κB [30]. In mice, EGCG (0.3%) directly inhibited T cell proliferative response to polyclonal and antigen-specific stimulation [41]. In 1-methyl-4-phenyl-1,2,3,6-tetrahydropyridine (MPTP)-induced Parkinson’s disease (PD) mouse model, EGCG could increase the ratio of CD3+CD4+ to CD3+CD8+ T lymphocytes and modulate peripheral immune response [42]. In human primary T cells, EGCG (10 μM or 20 μM) tended to inactivate AP-1 DNA-binding activity, and decreased INF-γ levels by 31.3% and 34.7%, IL-2 levels by 26.0% and 38.8%, IL-4 levels by 41.5% and 55.9%, as well as TNF-α levels by 23.0% and 37.6%, respectively [43]. Moreover, the level of p-JNK and p-ERK was also decreased by EGCG, but showing no effects to levels of p-p38 MAPK and total protein amounts [43].

Neutrophils are important in host defense and inflammation, though the latter might trigger and sustain several acute or chronic diseases. EGCG could inhibit metallo-elastase and serine-elastase secreted by macrophages and neutrophils, respectively [44,45]. EGCG could also reduce neutrophil transmigration through monolayers of endothelial cells [46]. In a transgenic zebrafish model, EGCG treatment reduced neutrophil response (accumulation, travel speed, and distance), downregulated the expression of IL-1β, TNF-α, and related signaling pathways [47]. Moreover, EGCG could inhibit apoptosis of activated neutrophils and chemokine-induced neutrophil chemotaxis *in vitro*, strongly inhibit neutrophil elastase, repress ROS activity, block neutrophil-mediated angiogenesis in vivo in an inflammatory angiogenesis model, enhance resolution in a pulmonary inflammation model, and significantly reduce consequent fibrosis [48]. GTPs inhibited TNF-α induction in macrophages through attenuating NF-kB activation [49]. In peritoneal macrophages, EGCG inhibited LPS-stimulated nitric oxide production and expression of iNOS through decreasing NF-κB activities [50]. In human peripheral blood mononuclear cells, EGCG could induce the production of neopterin, a marker of activated cellular immunity [51].

### 2.2. Effects on Humoral Immunity

GTPs and their derived substances could stimulate B-cell proliferation and antibody production effectively (Table 2). In BALB/c mice, tea polyphenols exerted stimulatory effects on humoral immune response through increasing number of antibody-secreted cells in spleen, and significantly increased the immunoglobulin M (IgM)-mediated and IgG-mediated immune response to non-particulate antigen (BSA) and particulate antigen (SRBCs) in a dose-dependent manner [52]. In particular, splenocytes reached the highest (344 ± 10/106) on day 14 postimmunization (*p*< 0.001) [52]. In case of polyphenols, the mice showed a sharp increase in IgM and IgG antibodies on days 14 and 21, respectively [52]. In addition, GTPs significantly reduced total IgG and type II collagen-specific IgG levels in serum and arthritic joints, as well as the neutral endopeptidase activity [53]. In vitro, EGCG could strongly enhance the direct plaque-forming cell (PFC) response to sheep red blood cells (SRBC) and show strong mitogenic activity towards mouse splenic B-cells [54]. In particular, the galloyl group on EGCG was responsible for enhancement [54]. ECG, EGCG, and theaflavin digallate (TFDG) displayed significant enhancement of the spontaneous proliferation of B-cells, though with quite different potencies [54]. In healthy Wuchang bream juveniles, dietary GTPs could elevate the content and mRNA levels of splenic IL-1β, TNF-α and IgM [55].

## 3. Immunomodulation through Anti-Inflammatory Mechanism

Inflammatory reactions are protective biological processes to eliminate harmful stimuli, which are performed by endogenous mediators, such as eicosanoids, oxidants, cytokines, chemokines, as well as lytic enzymes secreted by macrophages, neutrophils, and injured tissue itself [56,57]. Prostaglandins (PGs) and leukotrienes (LTs), critical factors involved in inflammatory responses, are produced in pathways catalyzed by cyclooxygenases (COX) and lipoxygenases (LOX) [58]. As a protective biological process, inflammation sometimes exerts detrimental outcomes in affected tissues, especially when reactive oxygen species (ROS) are produced. The highly reactive free radical nitric oxide (NO) can trigger toxic oxidative reactions, which will result in inflammation and tissue damage. Active PPARγ exerts anti-inflammatory roles through suppressing the expression of a wide range of pro-inflammatory genes (*IL-1β*, *IL-6*, *TNF-α*, *iNOS*, and *MMP-9*) in various cell types [59]. PPARγ activation could reduce inflammation through suppressing NF-κB activity, decreasing IKBα phosphorylation, covalently modifying NF-κB subunits for abrogated NF-κB-DNA interaction, and inducing NF-κB nuclear export [59].

The beneficial effects of GTPs and EGCG have been observed in animal models of rheumatoid arthritis (RA) [60], chronic inflammatory bowel diseases [61], lupus nephritis [62], and multiple sclerosis (MS) [63]. GTPs modulate neutrophil-mediated- and lymphocyte-mediated inflammation in IL-2-deficient mice [33,48]. In male DBA/1 mice (H-2q), GTPs significantly reduced the incidence of arthritis (33% to 50%), and reduced inflammatory mediator expression in arthritic joints, including COX2, IFN-γ, and tumor necrosis factor [53]. Diets supplemented with GTPs significantly attenuated inflammation through reducing the increment of IL-1, which was mainly produced by mononuclear macrophage [36]. Moreover, GTPs-supplementation also reduced pro-inflammatory cytokine IFN secreted by Th1 cell through inhibiting the activities of IkB kinase (IKK) and NF-κB [36]. In NOD mouse model for Hashimoto’s thyroiditis, GTPs administration led to an enhanced production of IL-10 by concanavalin A-stimulated splenocytes without interfering with thyroiditis development and serum thyroxine levels [64].

**Figure 2:**
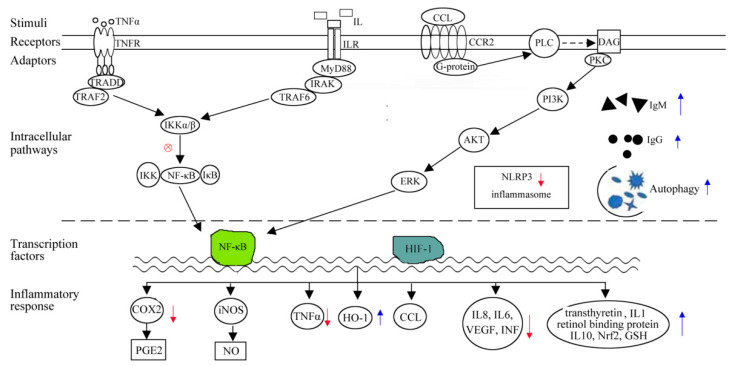
Schematic illustrating the immunomodulation mechanisms of GTPs and EGCG through anti-inflammatory mechanism.

EGCG could inhibit infiltration of inflammatory leukocytes [28]. The expression of proinflammation IL-8 could also be inhibited by EGCG [65]. In MPTP-induced PD mouse model, EGCG treatment could effectively reduce the expression of inflammatory factors TNF-α and IL-6 in serum [42]. In dental pulp cells pre-treated with LPS or PG, ECG could effectively inhibit the production of IL-6 and IL-8 [66]. In HDPC cells, ECG mitigated pulpal inflammation mostly through reducing LPS-/PG-mediated VEGF production, and inhibiting COX-2 expression in a dose-dependent manner [67]. In the human hepatoma cell line HepG2, ECG significantly reversed IL-6, reduced synthesis of negative acute-phase protein transthyretin and retinol binding protein, and finally enhanced host defense mechanisms towards inflammation [68]. ECG showed anti-inflammatory effects in HGFs mainly via preventing IL-17A-mediated CC chemokine ligand-20 (CCL20) production, inhibiting activities of p38 MAPK and ERK, as well as attenuating IL-17 receptor expression [69]. In LPS- induced macrophages and endotoxemia, ECG showed anti-inflammatory effects through inducing Nrf2/ARE-driven GSH and HO-1 levels, interfering with NF-κB and Nfr2/ARE transcriptional activities, and even suppressing MAPKs and PI3K/Akt signaling pathways [70]. ECG could intracellularly interact with Kelch repeat domains of Keap1, bind to extra cellular LPS, promote nuclear accumulation of Nrf2 proteins, blockade ERK1/2 and Nrf2/ARE signaling pathways, and ultimately attenuate pathological syndromes of LPS-induced sepsis and systemic inflammation (Figure 2) [21,70].

In an acute gout mouse model, oral administration of EGCG effectively alleviated gout inflammatory symptoms in foot tissue injected with monosodium urate (MSU) crystals, inhibited the de novo synthesis of mitochondrial DNA, and induced the production of ROS in primary macrophages [71]. Moreover, the in vivo suppressive effects of EGCG in mouse foot tissue were closely correlated with the suppression of NLRP3 inflammasome [71]. In mouse with gout disease, EGCG could suppress the activation of NLRP3 inflammasome in macrophages through blocking mitochondrial DNA synthesis, which contributed a lot to the prevention of gouty inflammation [71]. The inhibitory effects of EGCG on the NLRP3 inflammasome make EGCG a promising therapeutic option for NLRP3-dependent gout diseases, a chronic inflammatory disease evoked by the deposition of MSU crystals in joint tissues [71]. Oral or intravenous infusion of EGCG could reduce mRNA and protein expression of renal NLRP3 in lupus nephritis mouse model and contrast-induced nephropathy rat model, possibly leading to the reduction of NLRP3 inflammasome activation [61,72]. In mice with PFDA-induced liver damage and inflammation, GTPs or EGCG extended the survival time, inhibited weight loss, improved hepatic oxidative stress, cell apoptosis, steatosis, edema, necrosis, and degeneration, reduced hepatic inflammation and NLRP3 inflammasome activation [73].

## 4. Immunomodulation through Antioxidation Mechanism

Uncontrolled oxidative stress serves as the molecular basis of several immune-impaired pathologies [74]. Biotic and abiotic stressors, such as disease and chemical toxins, can result in over accumulation of ROS beyond the scavenging capability of the body, which is known as oxidative stress [74,75]. Particularly, the imbalance between generation of ROS and antioxidant defense capacity of body is closely associated with many dysfunctions, such as impaired immunity [75]. If immune cells are exposed to deleterious infection, they usually release ROS to eliminate exogenous invaders, and the ROS level is of physiologic relevance and very important for cell protection [37]. At the same time, the cumulative ROS may render host subjecting to oxidative stress and impair host T cell processes [76]. Oxidative stress is also blamed due to mediating diseases characteristic of immunodeficiency, including kidney failure, hypertension, ischemic-reperfusion injury, and alcoholic liver disease [77,78,79]. ROS, such as free radicals, oxygen ions, and peroxides, are implicated in cell damage and vital for host defense, which are frequently associated with inflammation [76].

GTPs have a special structure of polycyclic aromatic hydrocarbons, which can reduce oxidative free radicals and prevent critical cellular components from being oxidized [80]. Based on research in nutritional immunology, dietary GTPs are effective in meliorating immune related diseases through attenuating oxidative stress [36]. In piglets subjected to oxidative stress, dietary GTPs could promote proliferation and activation of T lymphocytes, elevate the ratio of CD4+/CD8+, attenuate the level of pro-inflammatory IL-1, decrease the concentration of serum IFN-*γ*, enhance serum concentrations of anti-inflammatory cytokine IL-4, lead the immune shift from Th1 to Th2, and alleviate growth depression, which showed significantly immunomodulatory potentials [36]. In juvenile black carp *Mylopharyngodon* *piceus*, GTPs (25-500 mg/kg) remarkably increased the content of serum superoxide dismutase (SOD) and glutamic oxalacetic transaminase (GOT), but decreased the contents of glutathione (GSH), glutathione peroxidase (GSH-Pox), malondialdehyde (MDA), cortisol, triglyceride (TG) and low-density lipoprotein cholesterol (LDL-C) at significant level (p <0.05) [80]. GTPs could effectively improve the growth performance and oxidative capacity on juvenile black carp with the optimal dosage of 50 mg/kg [80]. In healthy Wuchang bream juveniles, dietary GTPs have elevated the total antioxidant capacity through increasing contents of catalase and glutathione peroxidase in serum before and after ammonia exposure and reduce serum cortisol level [55]. In sleep disordered breathing rodents, GTPs could mitigate intermittent hypoxia-induced oxidative stress through reducing lipid peroxidation and decreasing the level of PGE2 [81].

ECG could catalyze oxidative DNA degradation in lymphocyte cell nuclei through mobilization of nuclear copper and induction of ROS production [82]. In dental pulp cells pre-treated with LPS or PG, ECG could effectively inhibit IL-6 and IL-8 production [66]. In HDPC cells, ECG could mitigate pulpal inflammation via reducing LPS/PG-mediated VEGF production, as well as inhibit COX-2 expression [66].

## 5. Benefits on Human Microbiota and Corresponding Immunological Implications

Green tea polyphenols could strongly reduce the body fat content, as well as hepatic triacylglycerol and cholesterol accumulation. In particular, the reduction was negatively correlated to the amount of Akkermansia and the total amount of intestinal bacteria [83]. A dysfunctional gut microbiota might participate in the pathogenesis of type 2 diabetes [83]. In C57BL/6J mice, green tea powder in combination with *Lactobacillus plantarum* could promote growth of *Lactobacillus* in the intestine, improve the diversity of intestinal bacteria, and attenuate high fat diet-induced inflammation [83]. In volunteers who did not usually consume green tea, green tea consumption acted as a prebiotic and improved the colon environment by increasing the proportion of the *Bifidobacterium* species [84]. Fermented green tea extract restored the changes in gut microbiota composition, including the ratios of *Firmicutes/Bacteroidetes* and *Bacteroides/Prevotella*, which is closely related with the development of obesity and insulin resistance [85]. In has been speculated that the fermented green tea extract improved obesity and other associated symptoms through modulating composition of gut microbiota, as mRNA expression levels of lipogenic and inflammatory genes were significantly downregulated in the white adipose tissue of mice [85]. Based on the high-throughput MiSeq sequencing and multivariate statistical analysis, green tea infusion consumption substantially increased diversity and altered the structure of gut microbiota in high-fat-diet induced obese C57BL/6J mice, which further exerted anti-obesity and anti-inflammatory activities [86]. Diversity of the total bacterial community reached the maximum after GTP treatment for almost 3 weeks, and then decreased when the mice were fed without GTP [87]. In particular, the relative abundance of *Bacteroidetes* increased from 0.56 ± 0.06 (1st week) to 0.60 ± 0.05 (3rd week), but *Firmicutes* decreased from 0.42 ± 0.06 (1st week) to 0.37 ± 0.02 (3rd week) [87]. Interestingly, *Bacteroidetes* and *Proteobacteria* still increased, but *Firmicutes* decreased even when the mice were fed without GTP (4th week) [87]. GTP could benefit the stability of certain gut microbiota in an environment-triggered microbial imbalance situation, providing prebiotic-like activity contributing to anti-obesity and anti-inflammatory effects. GTPs could boost mammal energy conversion by modulating gut-microbial community structure, gene orthologs, and metabolic pathways [88]. Following the increase of beneficial microbials in families *Clostridia, Ruminococcaceae*, *Lachnospiraceae*, and *Bacteroidaceae*, metabolic modulation could also been achieved through enriching many gene orthologs [88]. In rats, GTPs could enhance energy conversion through boosting mitochondrial TCA cycle and urea cycle of gut-microbiota [88]. Based on HILIC-HESI-tandem LC–MS, remarkable changes of 39 metabolites in the mitochondrial TCA cycle and urea cycle were observed, which showed significant dose- and time-dependencies on the GTPs treatment (0–1.5% wt/vol) [88].

## 6. Benefits towards Immune-Related Disease

GTPs can prevent many types of chronic diseases if regularly ingested in diet due to antioxidant, anticancer, anti-inflammatory, antiatherogenic, antidiabetic, antibacterial, and antiviral activities [89]. Moreover, GTPs exert protective effect on autoimmune diseases [89,90].

### 6.1. Benefits in Autoimmune Diseases

Besides genetic or acquired defects in immune tolerance or immune regulatory pathways, molecular mimicry to viral or bacterial proteins and impaired clearance of apoptotic cell materials are all the mechanisms of autoimmunity. Therefore, autoimmunity might be mostly due to copious production of autoantibodies and autoreactive cells. The autoimmune diseases could be classified into systemic and organ specific [91]. Autoimmune disorders may be caused by multiple interactions predisposition, and environmental triggers also contribute a lot to the autoimmune diseases [91,92]. GTPs have showed significantly therapeutic potential in a variety of autoimmune diseases.

Sjogren’s syndrome (SS), a relatively common autoimmunedisease, is characterized by inflammatory cell infiltration and loss of function of the lacrimal and salivary glands, which will result in ocular and oral health problems [92]. Selective inhibition of glandcell apoptosis, autoantigen expression, and production of pro-inflammatory cytokines are the three potential strategies for ameliorating SS [92]. Glandular cells may be important in initiating and sustaining SS, and the T-cell-mediated cytotoxicity and autoantibodies are also critical in the loss of gland function. Moreover, glandular epithelial cells contribute a lot to autoimmune process through secreting pro-inflammatory cytokines [91]. Aberrant expression and translocation of nuclear auto-antigens onto acinarcell membrane during apoptosis occurred, where they will be exposed to APCs (macrophages and dendritic cells) [93]. Furthermore, the auto-antigens redistributed in glandular cells and formed apoptotic bodies and blebs, around which autoantigen proteins (SS-A/Ro, SS-B/La, Ku, PARP, fodrin, golgins and NuMA) were clustered as subcellular structures [94]. The structural changes in auto-antigens may also contribute to the altered configuration of autoantigencluster, resulting in autoimmune response [94]. Naturally occurring phytochemicals in plant could be used in treating SS-associated disorders, such as GTPs, which could reduce the lymphocytic infiltration of submandibular gland in non-obese diabetic (NOD) mouse model for SS [95]. EGCG (0.2% consumption) protected NOD mouse submandibular glands from autoimmune-induced inflammation through reducing lymphocyte infiltration in the salivary glands during disease advancing stages, inhibiting apoptotic activity within the lymphocytic infiltrates, decreasing levels of serum total anti-nuclear antibody, and suppressing the expression of cell nuclear proliferation markers, such as proliferating cell nuclear antigen (PCNA) and Ki-67 [92].

Compared to a water-treated experimental autoimmune uveoretinitis (EAU) murine model, GTE attenuated clinical manifestations of uveitis, increased retinal-choroidal thicknesses (RCT) (1.100 ± 0.013 times vs1.005 ± 0.012 times, *P*< 0.001) and retinal vessel dilation (308.9 ± 6.189 units vs 240.8 units, *P*< 0.001) in a dose-dependent manner [96]. EGCG could also partially alleviate uveitic phenotypes and recover visualfunction in the murine model of EAU [96]. The treatment of GTE and its major component EGCG upregulated Th-17 associated pro-inflammatory genes, such as IL-1β, IL-6, IL-17A, and TNF-α [96]. GTE consumption serves as a potent therapeutic agent and a food supplement to develop alternative treatments against autoimmune uveitis [96].

### 6.2. Benefits in Cutaneousimmunity

UV light could affect skin biology and immune system, and lead to immunosuppression, premature aging, oxidative stress, and even carcinogenesis [1]. Immunosuppression induced by solar UV radiation (UVR) is a risk factor for melanoma and nonmelanoma skin cancers, which could potentially allow dysplastic cells being undetected and developing to neoplasms [97]. Notably, UV-induced DNA damage, particularly in terms of the formation of cyclobutane pyrimidine dimers (CPD), serves as critical molecular triggers for UV-induced immunosuppression [97]. As a 70-kDa heterodimer, IL-12 could induce Th1 responses and repair UV induced DNA damage [97]. The prevention of UVR induced immunosuppression by IL-12 depended on DNA repair and the induction of nucleotide excision repair enzymes [97]. UV radiation could mediate inflammatory and immunological reactions through activation of receptors, induction of DNA/RNA damage, and production of ROS [98]. UVR could activate multiple signaling cascades, including p38 MAPK, Jun N-terminal kinase, extracellular signal-regulated kinase 1/2, and NF-κB pathways in skin cells [98].

Human skin could be divided into epidermal, dermal, and hypodermal layers. In particular, epidermis consists of five layers, including stratum basale, stratum spinosum, stratum granulosum, stratum lucidum, and stratum corneum. Stratum basale, the innermost layer of epidermis, contains rapidly proliferating and differentiating keratinocytes, Merkel cells, and melanocytes. The stratum spinosum layer contains Langerhans cells, which serve as part of the immune system [98]. After UV exposure, an early inflammatory event soon takes place, which is characteristic by erythema and redness due to vasodilation of cutaneous blood vessels [98]. UV-induced TNF-α could diminish antigen presentation, reduce immunosurveillance, and initiate immunosuppression [98].

In mice fed with purified GTE, dose-dependent decrease in UVR-induced immunosuppression was observed, which were performed through contact hypersensitivity response (CHR) to 2,4-dinitrofluorobenzene [29]. Furthermore, the decrease in immunosuppression lasted 4 weeks, even after the resumption of a normal liquid diet [29]. In UV-irradiated mice, GTPs could reduce the migration of CPD positive cells to lymph nodes and improve nucleotide-excision repair mechanisms [29]. In human and animal studies, GTPs show significant protective effects against UV-induced skin damage and immunosuppression [1]. In keratinocytes cells exposed to UV radiation, IL-12 could enhance the NER enzyme activity [99]. DNA repair by topically applied EGCG might be through an IL-12-dependent mechanism [97]. Topical application of EGCG could effectively prevent UV-induced immunosuppression in rice through IL-12-dependent DNA repair, showing chemopreventive activity in prevention of photocarcinogenesis [97]. In particular, EGCG reduced number of CPDs+ cells and the migration of CPD+ APCs from skin to draining lymph nodes [97]. EGCG could prevent UV-induced immunosuppression by enhancing the levels of IL-12 [97]. Oral GrTP significantly delayed pathogenic leukocyte entry into skin following UV-B treatment [28].

### 6.3. Benefits towards Obesity-Related Immune Disease

Obesity is a major public health problem and global epidemic associating with comorbidities, such as diabetes, dyslipidemia, hypertension, cardiovascular diseases, and some cancers [100,101,102]. Strong relationship existed between adipose tissue and immune cells, as pro-inflammatory cytokines could not only increase free fatty acid (FFA) levels, but also induce ROS production [102]. Moreover, pro-inflammatory cytokines contributed a lot to the pro-inflammatory microenvironment in obesity and promoted activation and infiltration of immune cells into adipose tissue [102]. In obesity with high levels of hormones and nutrients, chronic low-grade inflammation state often present systemically or partially within the white adipose tissue (WAT) [103]. In particular, adipose tissue inflammation play important role in pathogenesis of diabetes. The energy-rich environment in obesity could damage immune cells present in blood stream and peripheral tissues [104]. Due to over-activation of immune cells, obese individuals are more subject to chronic inflammatory diseases, which would further destroy the functionality of immune cells and cause certain inflammatory response [105]. The increase in pro-inflammatory cytokines (IL-6, IL-1β, and TNF-α) and leptin released by adipocytes would drive lymphocytes to a Th1 phenotype in WAT [89]. Obesity often provides danger signals mimicking bacterial infection, which will drive a shift in M1 macrophages, CD8+, and CD4+ to Th1, Th2, and Th17, respectively. On the contrast, regulatory T cell (Treg) antiinflammatory lymphocyte numbers are decreased, which could prevent WAT inflammation and insulin resistance [106].

Significant immunomodulatory effects of GTPs under obese conditions have been observed, which were performed through recovering H_2_O_2_ and HOCl production, as well as reducing the levels of inflammatory cytokines [102]. In obese rats, GTPs could improve neutrophil function through increasing migration capacity of neutrophil, enhancing MPO activity, reducing catalase, increasing GSH/GSSG ratio, elevating production of H_2_O_2_, HOCl and O_2_·^-^, as well as decreasing expression of TNF-α, IL-6, TLR4, IκK, CD11b [102]. In Male Wistar rats, GTPs could reduce ROS production, improve redox status, reduce production of pro-inflammatory IL-2, IL-6, IL-1β, TNF-α cytokines, elevate exoression of Foxp3, IRF4, and IL-10, and inhibit expression of TLR4 [89]. In obese rats, GTPs could decrease cell proliferation, and drive lymphocytes to a more anti-inflammatory than pro-inflammatory microenvironment [89]. GTPs reduced the contents of triacylglycerol, total cholesterol, and low-density lipoprotein cholesterol, as well as inhibited body andhepatic fat accumulation [107,108]. In juvenile black carp *Mylopharyngodon* *piceus*, GTPs (500 mg/kg) significantly reduced the HDL-C and LDL-C [80]. EGCG could limit lipid absorption and even lower plasma lipid levels in rats [109].

TLR4 plays critical roles in innate immunity through inducing inflammatory cytokine, and abnormal activation of TLR4 could induce obesity-induced inflammation [110]. As a 67-kDa laminin receptor, EGCG3”Me could suppress TLR4 expression via upregulating E3 ubiquitin-protein ring finger protein 216 (RNF216), and cyclic GMP played important roles in this process (Figure 1) [110]. Moreover, EGCG3”Me could significantly attenuate TLR4 expression in adipose tissue, inhibit high-fat/high-sucrose (HF/HS)-induced upregulation of TNF-α in adipose tissue, and increase the serum monocyte chemoattractant protein-1 [110].

## 7. Problems and Prospects

Innate immunity and its molecular targets play important roles in the inflammatory response and protection against pathogens. The eukaryotic NF-κB transcription factors are critically involved in inflammation [36,59]. Interestingly, TLRs are expressed not only on immune cells but also on tumor cells constitutively or inducibly, thus natural products provide a means of dual targeting to combat diseases. Green tea is one of the most popular beverages around the world [22]. Epidemiological studies have suggested that the consumption of green tea is associated with reduced risk of some cancers, cardiovascular diseases, infections, and immunoregulation [111]. Green tea is rich in antioxidants that are useful to prevent onset and severity of arthritis [53,60]. In particular, GTPs possess significantly anti-inflammatory properties and are effective in inhibiting autoimmune diseases. Dietary GTPs could enhance immune responses to alleviate oxidative stress and damage caused by ammonia, showing potentials as a preventive or therapeutic measure in ammonia-exposed fish [55]. The increased levels of glutathione area critical mechanism underlying the beneficial effects of GTPs against some inflammatory disorders, and other mechanisms may also have a significant contribution. GTPs could not only divert immune types, but also reduce the burden of T lymphocytes under oxidative stress [36]. The great potential of GTPs to stimulate Th immune response against exogenous antigens makes them ideal as vaccines against pathogens and cancer cells. Oral therapy with GTPs could effectively prolong transplant survival and inhibit transplant-reactive T cell function in vivo, but the mechanisms need to be further discussed.

EGCG and ECG, the most abundant polyphenolic compound, possess effectively hepatic- and immune-protective properties against restraint stress through the combination of anti-oxidant, anti-inflammatory, and immunomodulatory activities [112]. EGCG could significantly reduce the release of stress hormones, which weak the restraint stress response [112]. Moreover, EGCG exerted neuroprotective effects in MPTP-induced PD mice model through modulating peripheral immune response, as EGCG treatment could restore the movement behavior of mice impaired by MPTP, and effectively protect tyrosine hydroxylase-positive cells in substantia nigra pars compacta region [42]. Accessory cells (mainly macrophages and DC) play critical roles in facilitating T cell activation and consequent proliferation, but EGCG could affect the function of accessory cells present in total splenocytes [41]. In restraint-challenged mice, EGCG (10-40 mg/kg) could significantly weak restraint stress response via preventing the release of H_2_O_2_, NOS and 8-isoprostane, reducing the levels of IL-1β, IL-2 and IL-6, normalizing the level of cytochrome P450 (CYP450) 1A2, 2D22, 2E1 and 3A11, relieving the inhibition status of T cells subsets in serum and IgA in BALF, as well as improving hepatic damage through decreasing the serum levels of alanine aminotransaminase (ALT) and aspartate transaminase (AST) [112]. EGCG could significantly delay the onset of autoimmune diabetes and effectively protect salivary gland cells from autoimmune-induced damage at multiple levels, inferring that EGCG could be used to delay and manage SS-like autoimmune disorders. In addition, EGCG could inhibit NF-κB activation, activator of transcription 1 (STAT1)-dependent cellular events, T cell proliferation, and cytokine production [33,40]. Moreover, EGCG could interfere with maturation and functions of DCs, as well as induce Treg differentiation [30,113]. In particular, the phenol rings of EGCG and ECG, comprising phenyl and hydroxyl group structures, possess significant anti-inflammatory, immunomodulatory, and antioxidant properties [90]. Oral consumption of EGCG could protect secretory cells against autoimmune-induced damage and sustain cellular function in secretory glands via preventing cells from signals for proliferation or apoptosis.

Although the association between natural products and immunity is well established and immune-related therapeutic potential cannot be underestimated, the exact molecular pathways and cellular mechanisms of immunomodulatory effects of GTPs are not fully understood. As a potent antioxidant to scavenge cytotoxic ROS, little is known about how GTPs protect immune cells and modulate immune response to oxidative stress in farm animals, as well as the precise mechanisms for the multi-level protection by EGCG. However, adverse effects of oral consumption of GTPs have been demonstrated in several studies [114]. Thus far, the effects of EGCG on T cell-associated functions have been mostly studied in specific disease animal models in vivo, but few were performed on healthy hosts [115,116,117,118,119]. The effect of GTPs towards transplant-reactive T cell immunity suggest that oral intake of green tea could serve as an effective adjunctive therapy to prevent transplant rejection in humans. However, further studies deeply evaluating the immunosuppressive actions of GTPs are beneficial for clinical trials aimed at inhibiting T cell immune responses directed at transplant antigens in humans. The concentration of polyphenols present in crude plant preparations could only exert an adjuvant effect for a short period, therefore high dose is needed for sustained activity.

Compared with individual compounds, green tea extracts showed more great health- promoting potential in due to the synergistic effects of different compounds. Up to now, great efforts have been made both in vitro and in vivo, but the accurate mechanisms are still unclear. Therefore, more carefully-designed studies are needed to deeply elucidate the immune-potentiating properties towards cellular and humoral immune responses. Moreover, directions involve clarifying the precise action mechanisms of green tea polyphenols, the optimal therapeutic doses, duration of treatment, and effects in both in vitro and in vivo model systems, especially in the setting of inflammatory diseases, are also needed to be performed. Whether or not GTPs act on TLRs through covalent or non-covalent binding needs to be deeply clarified. Due to potential safety profiles and immunomodulatory effect, green tea might provide a new source for chemopreventive or therapeutic agents for various chronic diseases.

## Figures and Tables

**Table 1:** TLR subfamilies and corresponding ligands.

TLR Subfamilies	Recognized Ligands
TLR1, TLR2, TLR6	lipopeptides and glycolipids
TLR7, TLR8, TLR9	nucleic acids (ssRNA and unmethylated CpG)
TLR3	dsRNA associated with viral infection
TLR4	fibronectin, lipopolysaccharides (LPS), and heat shock proteins
TLR5	bacterial flagellin
TLR11, TLR12	profilin and actin-binding protein

**Table 2:** The immune-potentiating effects of tea polyphenols.

Component	Model	Effect	Reference
GTPs and their derivatives	Human cells	Stimulation of g multiple TLR signaling pathways	[24]
	Murine lymphocytes	Inhibition of proliferation	
EGCG	RAW 264.7 cells (a murine monocytic cell line, ATCC TIB-71)	Inhibition of IKKβ and NF-κB activation	[26]
	293T (human embryonic kidney cells)		
EGCG	Human PBMC cells	Inhibition of the production of IFNγ	[27]
EGCG	C3H/HeN mice	Inhibition of the depletion of APCs and UV-induced immunosuppression	[28,29]
EGCG	Mouse bone marrow-derived DCs	Inhibition of DCs maturation	[29,30]
	Human monocytes-derived DCs		
EGCG	Kuruma shrimp *Marsupeneaus japonicus* challenged with WSSV	Stimulation of innate immune-related gene expressions (*IMD*, *proPO*, *QM*, *myosin*, *Rho*, *Rab7*, *p53*, *TNF-alpha*, *MAPK*, and *NOS*)	[32]
	Gram-negative bacterium *Vibrio alginolyticus*		
EGCG	shrimp innate immune system	Enhance of immune parameters, inhibition of apoptosis	[32]
GTPs	Interleukin-2-deficient mice, intestinal epithelial cell line IEC-6	Inhibition of IFNγ-induced phosphorylation and function of STAT1; inhibition of iNOS and NF-κB upregulation; inhibition of transcription and secretion of TNFα and IFNγ	[33,34,35]
GTPs	MDAmb231, MDAmb468, MCF7, Hela, HepG2	Inhibition of IFNγ-induced phosphorylation and function of STAT1	[35]
GTPs	Piglets	Elevation of antiinflammatory cytokine IL-4; inhibition of the ratio of IFNγ/IL-4; stimulation of immune cell proliferation and T lymphocytes; elevation of CD4+ T cell percentage and the ratio of CD4+/CD8+; improvement of LTT	[36]
GTPs	Lymphocytes	Inhibition of IFNγ secretion; stimulation of T cells and APCs apoptosis; inhibition of T cell division and cycle progression in a dose-dependent manner	[38]
GTPs	T lymphocytes	influencing expression of T-cell receptor complex and antigen-specific T-cell responses	[39]
EGCG	Spleen cells isolated from C57BL mice	Inhibition ofsplenocyte proliferation, T cell division, and cell cycle progression	[40]
EGCG	murine DCs	Inhibition of MAPK and NF-kB	[30]
EGCG	MPTP-induced Parkinson’s disease mouse	Increase of the ratio of CD3+CD4+ to CD3+CD8+ T lymphocytes; modulating peripheral immune response	[42]
EGCG	Macrophages and neutrophils	Inhibition of metallo-elastase and serine-elastase	[43,44]
EGCG	Human umbilical vein endothelial cells	Inhibition of neutrophil transmigration	[45]
EGCG	Transgenic zebra fish	Inhibition of neutrophil response (accumulation, travel speed, and distance) expression of IL-1β and TNFα, as well as related signaling pathways	[46]
EGCG	Inflammatory angiogenesis model, pulmonary inflammation model	Apoptosis inhibition of activated neutrophils and chemokine-induced neutrophil chemotaxis; inhibition of neutrophil elastase, ROS activity, neutrophil-mediated angiogenesis, and fibrosis	[47]
GTPs	Macrophages	Inhibition of TNF-α induction and NF-kB activation	[48]
EGCG	peritoneal macrophages	Inhibition of LPS-stimulated NO production, iNOS expression, and NF-kB activities	[49]
EGCG	Human peripheral blood mononuclear cells	Inducement of neopterin production	[50]
Tea polyphenols	BALB/c mice	increase of antibody-secreted cells in spleen and IgM-mediated IgG-mediated immune response	[51]
GTPs	Mice	Decrease of total IgG and type II collagen-specific IgG levels in serum and arthritic joints, as well as the neutral endopeptidase activity	[52]
EGCG	Mouse	Enhancement of PFC response to sheep red blood cells, strong mitogenic activity towards splenic B-cells	[53]
GTPs	Wuchang bream juveniles	Elevation of content and mRNA levels of splenic IL-1β, TNFα and IgM	[54]

## Data Availability

Not applicable.

## Abbreviations

Ag	Antigen
ALT	Alanine aminotransaminase
APCs	Antigen-presenting cells
AST	Aspartate transaminase
BSA	Bovine serum albumin
CCL20	CC chemokine ligand-20
CHR	Contact hypersensitivity response
CPD	Cyclobutanepyrimidine dimers
COX	Cyclooxygenase
Con A	Concanavalin A
CYP450	Cytochrome P450
DCs	Dendritic cells
EAU	Experimental autoimmune uveoretinitis
EGCG	(–)-Epigallocatechin-3-gallate
EGCG3”Me	(–)-Epigallocatechin-3-O-(3-O-methyl) gallate
ECG	(–)-Epicatechin gallate
ERK	External signal-regulated kinases
FFA	Free fatty acid
GOT	Glutamic oxalacetic transaminase
GTE	Green tea extracts
GTPs	Green tea polyphenols
GSH	Glutathione
GSH-Pox	Glutathione peroxidase
HESI	Heated electrospray ionization
HDPC cells	Human dental pulp-derived cells
HDL-C	High-density lipoprotein cholesterol
HGFs	Human gingival fibroblasts
HILIC	Hydrophilic interaction liquid chromatography
HO-1	Heme oxygenase-1
HF/HS	High-fat/high-sucrose
Ig	Immunoglobulin
IgM	Immunoglobulin M
IFNγ	Interferon gamma
IL	Interleukin
IKKβ	IkB kinase
iNOS	Inducible nitric oxide synthetase
IRAKs	IL-1R associated kinases
LC-MS	Liquid chromatography mass spectrometry
LDL-C	Low-density lipoprotein cholesterol
LOX	Lipoxygenase
LPS	Lipopolysacharrides
LTs	Leukotrienes
LTT	T lymphocyte transformation
MAPK	Mitogen-activated protein kinase
MDA	Malondialdehyde
MHC	Major histocompatibility complex
MMP	Matrix metalloproteinase
MPTP	1-Methyl-4-phenyl-1,2,3,6-tetrahydropyridine
MSU	Monosodium urate
MS	Multiple sclerosis
MyD88	Myeloid differentiation primary response gene 88
NF-κB	Nuclear factor-κB
NLRP3	NOD-like receptor protein 3
Nk	Natural killer cells
No	Nitric oxide
Nod	Non-obese diabetic
Pamps	Pathogen-associated molecular patterns
PARP	Poly(ADP-ribose) polymerase
PCNA	Proliferating cell nuclear antigen
PD	Parkinson’s disease
p-ERK	Phosphorylated extracellular signal-regulated kinase
PGE2	Prostaglandin E2
PFC	Plaque-forming cell
PGs	Prostaglandins
PI3K	Phosphatidylinositol-3-kinase
p-JNK	Phosphorylated c-Jun N-terminal
PPARγ	Peroxisome proliferator-activated receptor gamma
PRRs	Pattern-recognition receptors
RA	Rheumatoid arthritis
RCT	Retinal-choroidal thicknesses
RNF216	Ringfinger protein 216
ROS	Reactive oxygen species
SEB	Staphylococcus aureus enterotoxin B
SRBCs	Sheep red blood cells
SS	Sjogren’s syndrome
SOD	Serum superoxide dismutase
ssRNA	Single stranded RNA
TCA	Tricarboxylic acid
Th	T helper cell
TFG	Theaflavin digallate
TG	Triglyceride
TIR	Toll/interleukin 1 receptor
TLR	Toll-like receptor
Treg	Regulatory T cell
UV	Ultraviolet
UVR	Ultraviolet radiation
WSSV	White spot syndrome virus
WAT	White adipose tissue
